# Association of Dromedary Camels and Camel Ticks with Reassortant Crimean-Congo Hemorrhagic Fever Virus, United Arab Emirates

**DOI:** 10.3201/eid2709.210299

**Published:** 2021-09

**Authors:** Jeremy V. Camp, Pia Weidinger, Sathiskumar Ramaswamy, Dafalla O. Kannan, Babiker Mohammed Osman, Jolanta Kolodziejek, Noushad Karuvantevida, Ahmad Abou Tayoun, Tom Loney, Norbert Nowotny

**Affiliations:** Medical University of Vienna, Vienna, Austria (J.V. Camp);; University of Veterinary Medicine Vienna, Vienna (J.V. Camp, P. Weidinger, J. Kolodziejek, N. Nowotny);; Mohammed Bin Rashid University of Medicine and Health Sciences, Dubai, United Arab Emirates (S. Ramaswamy, N. Karuvantevida, A. Abou Tayoun, T. Loney, N. Nowotny);; Al Jalila Children’s Hospital, Dubai (S. Ramaswamy, A. Abou Tayoun);; Al Ain City Municipality, Al Ain, United Arab Emirates (D.O. Kannan, B.M. Osman)

**Keywords:** Crimean-Congo hemorrhagic fever virus, CCHFV, viruses, reassortant, dromedary camels, Camelus dromedarius, camels, Ixodidae, ticks, camel ticks, Hyalomma, vector-borne infections, transmission, zoonoses, United Arab Emirates

## Abstract

We previously detected a potentially novel reassortant of Crimean-Congo hemorrhagic fever virus in camels at the largest livestock market in the United Arab Emirates. A broader survey of large mammals at the site indicated zoonotic transmission is associated with dromedaries and camel ticks. Seroprevalence in cattle, sheep, and goats is minimal.

Crimean-Congo hemorrhagic fever virus (CCHFV) is a tickborne nairovirus (order *Bunyavirales*) that is maintained primarily in *Hyalomma* ticks (Ixodidae), and various mammalian livestock serve as amplifying hosts. Humans might become infected from the bite of an infected tick or during slaughter of a viremic animal, and the infection might lead to severe viral hemorrhagic fever and death. In the Arabian Peninsula, human cases are sporadically reported and seem to be primarily associated with abattoir work (*1*,*2*) or nosocomial human-to-human transmissions (*3*).

CCHFV is genetically diverse and has a relatively wide geographic distribution (*4*). The virus might be introduced into nonendemic regions through commercial trading of livestock (*5*) or through phoretic transport of ticks on migratory birds (*6*,*7*). Comparatively little is known about the zoonotic transmission of the virus in the United Arab Emirates or whether past outbreaks were only associated with recent importations (*5*).

We previously performed a cross-sectional virologic and serologic survey of CCHFV in dromedary camels (*Camelus dromedarius*) at various sites throughout the United Arab Emirates (*8*). We found the highest transmission activity at a large livestock market, in which viral nucleic acids were detected in camel ticks (*Hyalomma dromedarii*) and camels. On the basis of partial gene sequences from the small and medium (M) RNA gene segments, the virus strain appeared to be a novel reassortant (*8*). We performed a follow-up study at the same market to test whether other livestock are involved in the transmission of CCHFV and to better characterize the virus strain.

## The Study

During October 10‒24, 2019, we sampled camels, cattle, goats, and sheep upon their entry to a livestock market in the emirate of Abu Dhabi, United Arab Emirates (≈24.16°N, 55.81°E) (Appendix Figure 1). All procedures were conducted as part of standard veterinary inspection required for market entry.

We obtained 5 mL of blood from each animal, separated serum by centrifugation, and stored serum at ‒80°C. We tested serum for CCHFV-reactive antibodies by using a commercial kit (ID Screen CCHF Double Antigen Multi-species; IDvet, https://www.id-vet.com). Antibodies to CCHFV were found in 72/90 camels, 7/51 cattle, 1/45 goats, and 4/55 sheep (Table). We extracted total nucleic acids from 200 μL of the same serum samples by using a commercial kit (QIAamp Viral RNA Mini Kit; QIAGEN, https://www.qiagen.com) and QIAcube or QIAcube HT Extraction Robots (QIAGEN). We tested extracts for CCHFV RNA by using a commercially available quantitative reverse transcription PCR (a RT-PCR) assay (RealStar CCHFV RT-PCR Kit 1.0; Altona Diagnostics, https://www.altona-diagnostics.com). Viral nucleic acids were detected in 2 of 90 camels, and in no other species at the market.

**Table:** Evidence of exposure to Crimean-Congo hemorrhagic fever virus in animals at a livestock market, United Arab Emirates, 2019

Species	No. sampled	No. antibody positive	No. virus RNA positive
Camels	90	72	2
Cattle	55	7*	0
Goats	45	1	0
Sheep	55	4	0

*Serum was not available for 4 cattle.

During blood collection, we thoroughly searched each animal (≈2 min) and removed attached ticks. Ticks were frozen at −80°C, and adult ticks were morphologically identified by using various keys on an ice cold plate. We collected 210 *H. dromedarii* adults, 4 unidentified *Hyalomma* sp. adults, and 4 *Hyalomma* sp. nymphs from 84/90 camels. No ticks were found on any other animal during this sampling session, and it was later confirmed that topical acaricides were routinely used for all animals except camels, where they were applied only sporadically.

We processed frozen ticks to screen for viral nucleic acids by making a parasagittal section using a sterile scalpel and then made homogenized pools containing half-ticks (<5 per pool, pooled per tick species, and per individual host) in a bead mill in buffered saline before adding DNA/RNA Shield (ZymoResearch, https://www.zymoresearch.com) and performing nucleic acid extraction and qRT-PCR. We detected CCHFV RNA in 3 pools of *H. dromedarii* ticks taken from 2 camels, 1 of which was seropositive and the other seronegative.

We then extracted nucleic acids from the remaining halves of 3 ticks collected in the previous sampling session (April 2019) (*8*) and 2 ticks collected in this sampling session from pools that were positive by qRT-PCR. After confirming the presence of CCHFV nucleic acid in the individual tick halves, we processed the samples by using a shotgun transcriptomic sequencing approach (Appendix). In brief, we quality-filtered, trimmed, and assembled paired-end reads from Illumina (https://www.illumina.com) sequencing into scaffolds, which we then searched against the National Center for Biotechnology Information nonredundant database using blastn (https://blast.ncbi.nlm.nih.gov).

We identified near-complete genomes of CCHFV, including complete open reading frames of all but 1 gene segment (missing 577 nt from the large segment open reading frame 3′ mRNA end), from all 5 samples (GenBank accession nos. MW548490‒504) (Appendix). We aligned the sequences to selected reference sequences representing the major genotypes (*4*). All sequences had high identity to each other (98.8%–100%) and to a recently described CCHFV (98.5%–99.6%) identified in a dromedary from the same livestock market in the emirate of Abu Dhabi, but 4 years earlier, during 2015 (*9*) (Appendix Table). The M segment was the most variable; it had 52–64 nonsynonymous mutations compared with the sample obtained during 2015 from the same place, and 1–40 nonsynonymous mutations among the 5 sequences (Appendix Table). 

We constructed phylogenetic trees from the alignments of the respective gene segments by using maximum-likelihood analysis over 500 bootstrap replicates of the general time reversible plus invariant sites plus gamma distribution substitution model and 4 gamma categories. Small segments fit within the previously described genotype from Africa (group III sensu [4] and Africa 3 sensu [9]), and large segments had a common ancestor with sequences from Africa (groups I and III sensu [4], Africa 1/3 sensu [9]), and Europe (group V sensu [4] and Europe 1 sensu [9]) (Appendix Figures 2, 3). The M segment appeared to be a novel lineage of CCHFV (Figure).

**Figure:**
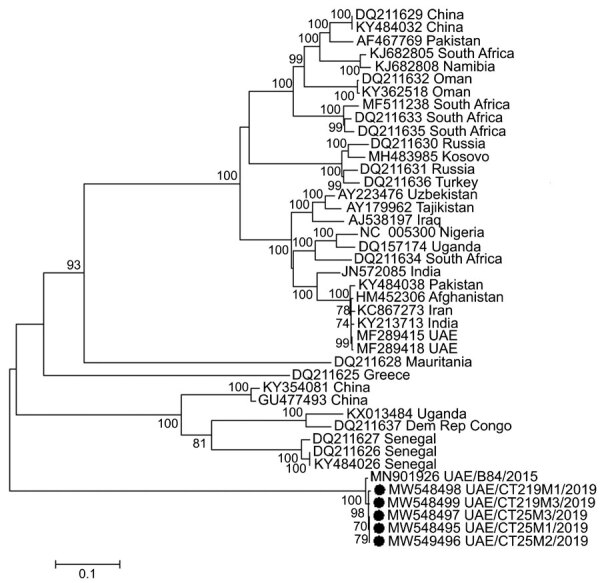
Molecular phylogeny of Crimean-Congo hemorrhagic fever virus medium RNA segments, United Arab Emirates, 2019 (solid circles), and reference viruses. Viruses from this study were obtained from camel ticks (*Hyalomma dromedarii*) removed from dromedary camels at a large livestock market in the emirate of Abu Dhabi. Other virus sequences included were selected as representatives of the major small and large RNA segment genotypes for which full-length sequences of all 3 viral genomic segments were available. Viruses listed include GenBank accession number and country of origin. Maximum-likelihood analysis of coding-complete sequences was performed by using the general time reversible plus invariant sites plus gamma distribution substitution model and 4 categories with >500 bootstrap replicates. Numbers along branches are percentage support, showing only values >65%, and branch length is relative to the number of substitutions per site, as indicated by the scale bar. Dem Rep Congo, Democratic Republic of the Congo; UAE, United Arab Emirates.

## Conclusions

We concur with the findings of Khalafalla et al. (*9*), who provided additional serologic and virologic evidence that the CCHFV strain in the United Arab Emirates might be specifically associated with camels and camel ticks. Our study differs from previous studies, in that our sampling was performed directly at entry to a livestock market, but our previous study was performed after camels had entered the market (range 0–77 d, mean 12.2 d). Moreover, all animals were raised in the United Arab Emirates, although some sheep and goats were imported as young animals from India, Saudi Arabia, and Oman. Combined, the evidence suggests that the CCHFV strain has spread throughout the United Arab Emirates, but the livestock market is also a focus of transmission (*8*).

Although this strain of CCHFV appears to be circulating at least since 2015 in the United Arab Emirates (*8*,*9*), there is additional evidence that it might be more widely distributed (*10*). Evidence of increased exposure of camels to CCHFV at livestock markets in contrast to other locations (e.g., private farms or in tourist/recreational use) increases the potential for the virus to be transported long distances through the camel trade (*8*). We support the suggestion of Khalafalla et al. to increase efforts to characterize CCHFV from camels, camel ticks, and other livestock in a broader geographic region (*9*). The infection of camels appears to be systemic; virus was detected in blood (*8*; this study) and the respiratory tract (*9*). The low CCHFV-reactive seroprevalence and low tick burden on other livestock entering the market is probably caused by use of acaricides, which are reportedly used only sporadically on camels. We therefore recommend increased vigilance, including use of acaricides on all livestock, including dromedaries, to limit spillover to humans involved in the camel trade, abattoir workers, and those handling raw meat or consuming raw camel milk.

Appendix Additional information on association of dromedary camels and camel ticks with reassortant Crimean-Congo hemorrhagic fever virus, United Arab Emirates.
