# Supplementary material for: Association of Dromedary Camels and Camel Ticks with Reassortant Crimean-Congo Hemorrhagic Fever Virus, United Arab Emirates
Source: Emerg Infect Dis. 2021 Sep;27(9):2471–4. doi: 10.3201/eid2709.210299 (PMC8386785; doi:10.3201/eid2709.210299)
Supplement: Appendix — Additional information on association of dromedary camels and camel ticks with reassortant Crimean-Congo hemorrhagic fever virus, United Arab Emirates. [file 21-0299-Techapp-s1.pdf]

Article DOI: https;//doi.org/10.3201/eid2709.210299

Association of Dromedary Camels and Camel Ticks with Reassortant Crimean-Congo Hemorrhagic Fever Virus, United Arab Emirates

Appendix

Supplemental Methods

Shotgun transcriptome sequencing was performed as described (*1*,*2*). Raw paired reads were preprocessed to remove adaptor sequences and filter out low-quality bases by using Illumina BCL2Fastq version 2.20 (https://www.illumina.com) and the Trimmomatic-0.36 program (*3*). Preprocessed, high-quality data were used for de novo assembly, and contigs obtained were then reassembled into scaffolds by using SPAdes-3.14.0 (*4*). These scaffolds were subsequently searched against a nonredundant database by using the BlastN program (*5*). We obtained ≈3 GB–7 GB of high-quality preprocessed data for all 5 isolates. We were able to assemble nearly entire large, medium, and small RNA segments by using de novo assembly (Appendix Table).

Contigs of interest matching Crimean-Congo hemorrhagic fever orthonairovirus were aligned with selected reference sequences from GenBank representing the major genotypes by using the MUSCLE algorithm (*6*) in MEGA7 (*7*) over 8 iterations with the unweighted pair group method with arithmetic mean clustering method.

Reference sequences are comprised of full-length sequences from which all 3 genetic segments are available. Phylogenetic trees of the small (Appendix Figure 2), medium (Figure 1 in main text), and large (Appendix Figure 3) segments were made from these alignments by using the general time reversible + invariant sites + gamma distribution substitution model, inferring branches with maximum-likelihood methods over 500 bootstrap replicates.

The assembled segments were also compared with the camel Crimean-Congo hemorrhagic fever virus described from the same livestock market in Abu Dhabi Emirates 4

years earlier during 2015 (*9*). We provide sequence identities for this reference isolate (Appendix Table).

References

1. Tayoun AA, Loney T, Khansaheb H, Ramaswamy S, Harilal D, Deesi ZO, et al. Multiple early introductions of SARS-CoV-2 into a global travel hub in the Middle East. Sci Rep. 2020;10:17720. PubMed https://doi.org/10.1038/s41598-020-74666-w

2. Harilal D, Ramaswamy S, Loney T, Suwaidi HA, Khansaheb H, Alkhaja A, et al. SARS-CoV-2 Whole genome amplificaton and sequencing for effective population-based surveillance and control of viral transmission. Clin Chem. 2020;66:1450–8. PubMed https://doi.org/10.1093/clinchem/hvaa187

3. Bolger AM, Lohse M, Usadel B. Trimmomatic: a flexible trimmer for Illumina sequence data. Bioinformatics. 2014;30:2114–20. PubMed https://doi.org/10.1093/bioinformatics/btu170

4. Bankevich A, Nurk S, Antipov D, Gurevich AA, Dvorkin M, Kulikov AS, et al. SPAdes: a new genome assembly algorithm and its applications to single-cell sequencing. J Comput Biol. 2012;19:455–77. PubMed https://doi.org/10.1089/cmb.2012.0021

5. Altschul SF, Gish W, Miller W, Myers EW, Lipman DJ. Basic local alignment search tool. J Mol Biol. 1990;215:403–10. PubMed https://doi.org/10.1016/S0022-2836(05)80360-2

6. Edgar RC. MUSCLE: multiple sequence alignment with high accuracy and high throughput. Nucleic Acids Res. 2004;32:1792–7. PubMed https://doi.org/10.1093/nar/gkh340

7. Kumar S, Stecher G, Tamura K. MEGA7: Molecular Evolutionary Genetics Analysis version 7.0 for bigger datasets. Mol Biol Evol. 2016;33:1870–4. PubMed https://doi.org/10.1093/molbev/msw054

8. Deyde VM, Khristova ML, Rollin PE, Ksiazek TG, Nichol ST. Crimean-Congo hemorrhagic fever virus genomics and global diversity. J Virol. 2006;80:8834–42. PubMed https://doi.org/10.1128/JVI.00752-06

9. Khalafalla AI, Li Y, Uehara A, Hussein NA, Zhang J, Tao Y, et al. Identification of a novel lineage of Crimean-Congo haemorrhagic fever virus in dromedary camels, United Arab Emirates. J Gen Virol. 2021;102:doi: 10.1099/jgv.0.001473. PubMed https://doi.org/10.1099/jgv.0.001473

Appendix Table. Summary of genomic sequences of Crimean-Congo hemorrhagic fever virus obtained from camel ticks by using a shotgun transcriptomic sequencing approach, United Arab Emirates, 2019*

GenBank Accession no.	Isolate name	Segment	Sequence length, bp	% nt identity†	No. aa changes†
MW548490	UAE/CT25M1/2019	S	1,670	99.6	1
MW548495	UAE/CT25M1/2019	M	5,302	98.8	63
MW548500	UAE/CT25M1/2019	L	11,316	99.2	11
MW548491	UAE/CT25M2/2019	S	1,663	99.6	1
MW548496	UAE/CT25M2/2019	M	5,324	98.8	64
MW548501	UAE/CT25M2/2019	L	12,114	99.2	12
MW548492	UAE/CT25M3/2019	S	1,648	99.6	1
MW548497	UAE/CT25M3/2019	M	5,301	98.8	63
MW548502	UAE/CT25M3/2019	L	12,105	99.2	12
MW548493	UAE/CT219M1/2019	S	1,656	99.6	2
MW548498	UAE/CT219M1/2019	M	5,309	99.0	52
MW548503	UAE/CT219M1/2019‡	L	11,924	99.2	9
MW548494	UAE/CT219M3/2019	S	1,663	98.5	11
MW548499	UAE/CT219M3/2019	M	5,286	98.8	64
MW548504	UAE/CT219M3/2019	L	12,122	99.2	8

*L, large; M, medium; S, small; UAE, United Arab Emirates.
†Reference sequences: Camel CCHFV/Abu Dhabi/B84, S: MN901927; M: MN901926; L: MN901925;
‡This sequence is not coding-complete; it is missing 577 nt from the 3′ complementary RNA end.

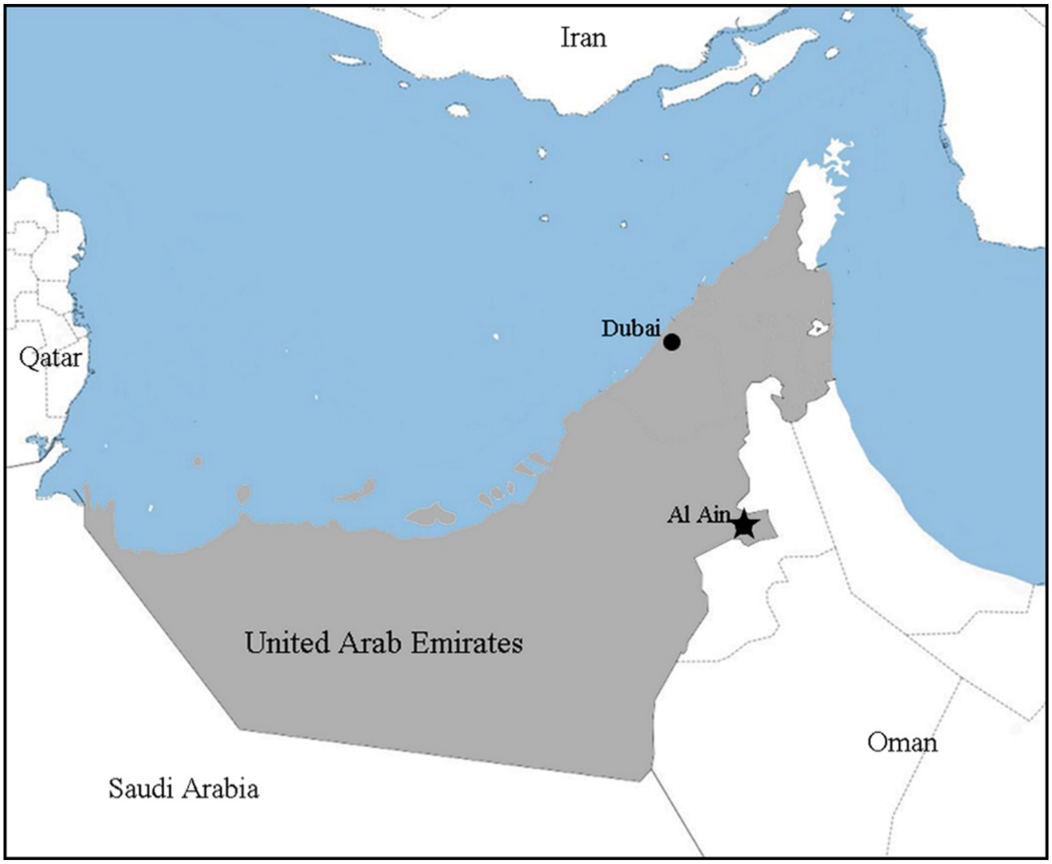

Appendix Figure 1. Map of the United Arab Emirates (gray), neighboring countries (white), and the Arabian Gulf (blue). Sampling was performed at a livestock market in Al Ain (black star) and in Dubai (black circle).

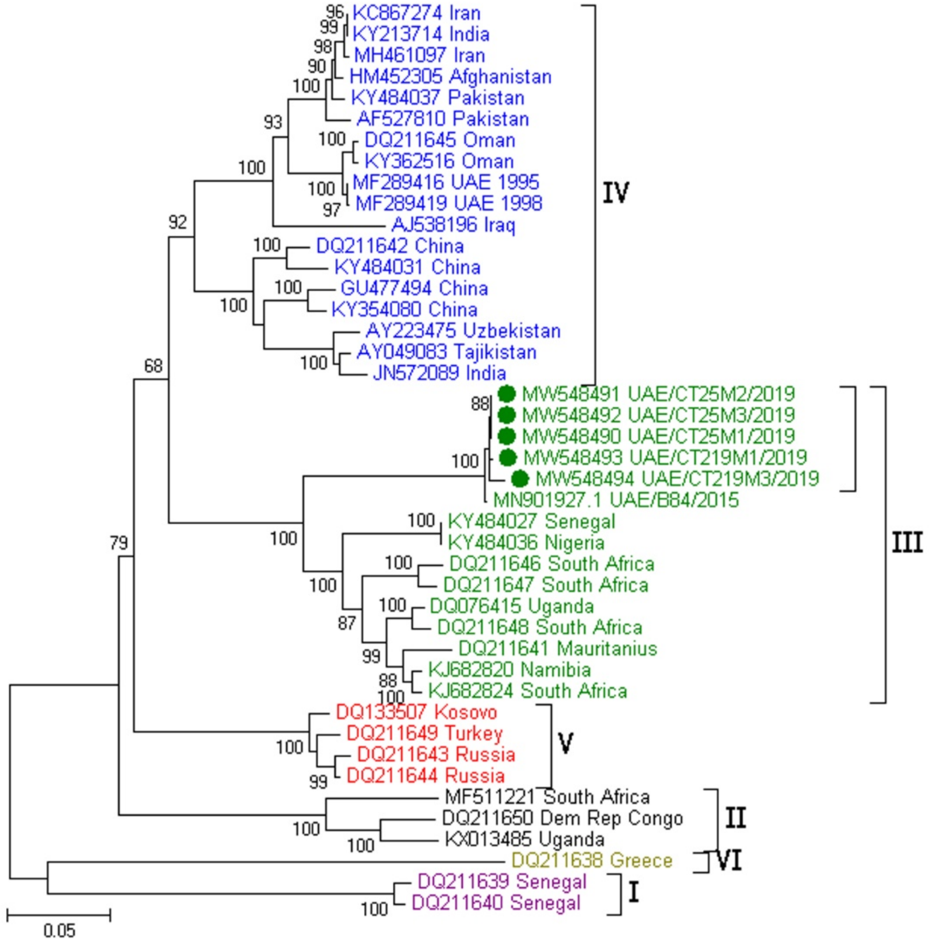

Appendix Figure 2. Molecular phylogeny of selected Crimean-Congo hemorrhagic fever virus small RNA segments. Viruses from this study are indicated by solid circles, and were isolated from camel ticks (*Hyalomma dromedarii*) collected from dromedary camels at a large livestock market in United Arab Emirates, 2019. Maximum-likelihood analysis of coding-complete sequences was performed by using the general time reversible + invariant sites + gamma distribution substitution model and 4 categories with >500 bootstrap replicates. Viruses include GenBank accession number and country of origin, and major lineages are indicated in brackets on the right and colored according to Deyde et al. (*8*). Numbers along branches are percentage support, showing only values >65%, and branch length is relative to the number of substitutions per site, as indicated by the scale bar. Dem Rep Congo, Democratic Republic of the Congo; UAE, United Arab Emirates.

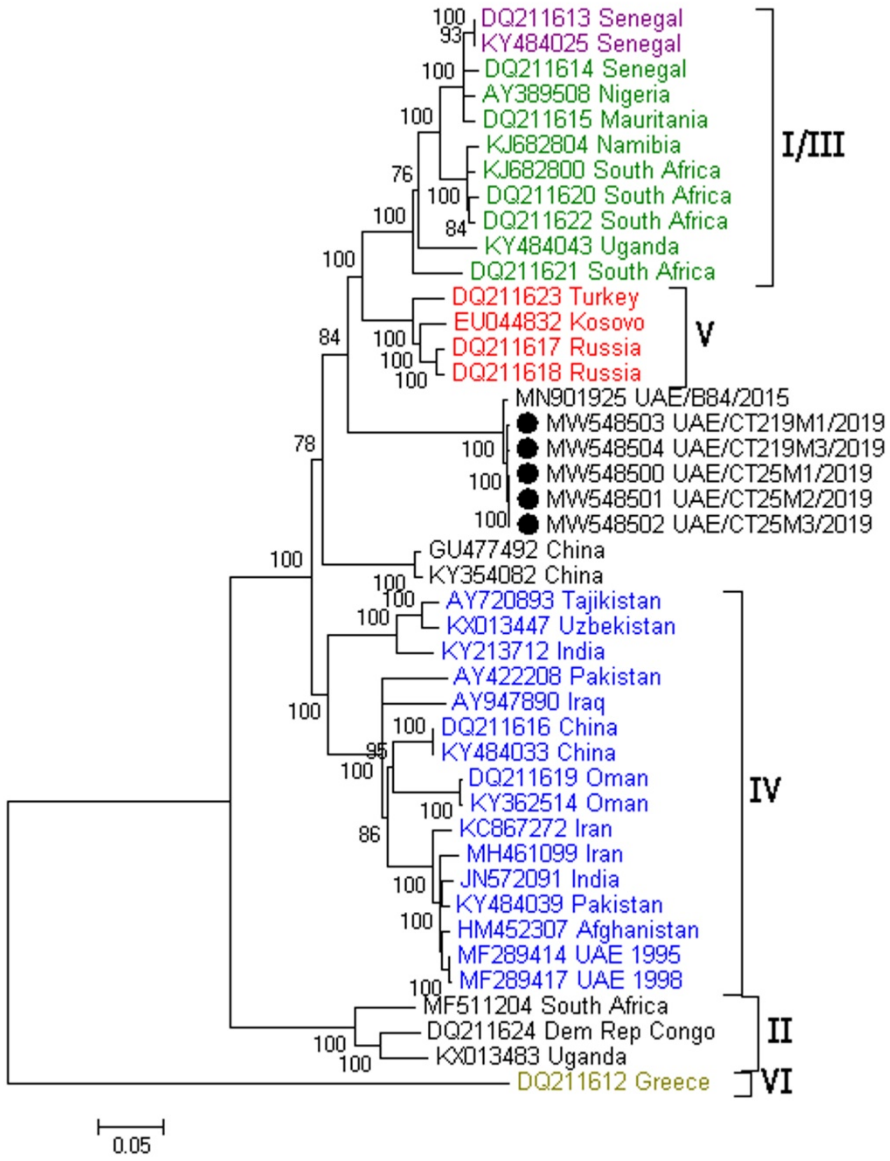

Appendix Figure 3. Molecular phylogeny of selected Crimean-Congo hemorrhagic fever virus large RNA segments. Viruses from this study are indicated by solid circles, and were obtained from camel ticks (*Hyalomma dromedarii*) collected from dromedary camels at a large livestock market in the emirate of Abu Dhabi, United Arab Emirates, 2019. Maximum-likelihood analysis of coding-complete sequences (with the exception of strain Al Ain/CT25M1/2019) was performed by using the general time reversible + invariant sites + gamma distribution substitution model and 4 categories with >500 bootstrap replicates. Viruses include GenBank accession number and country of origin, and major lineages are indicated in brackets on the right and colored according to Deyde et al. (*8*). Numbers along branches are percentage support, showing only values >65%, and branch length is relative to the number of substitutions per site, as indicated by the scale bar. Dem Rep Congo, Democratic Republic of the Congo; UAE, United Arab Emirates.
